# Editorial: Promoting replicability: empowering method and applied researchers in driving reliable results

**DOI:** 10.3389/fpsyg.2026.1835500

**Published:** 2026-06-18

**Authors:** María Paula Fernández-García, Guillermo Vallejo-Seco, Pablo Livácic-Rojas

**Affiliations:** 1 Department of Psychology, University of Oviedo, Oviedo, Spain; 2 Department of Psychology, University of Santiago de Chile, Santiago, Chile

**Keywords:** measurement invariance, methodological responsibility, Monte Carlo simulation, online data, replicability crisis, research integrity

The replicability crisis in the behavioral sciences should no longer be understood as a merely technical problem confined to the failure to reproduce specific findings. Rather, it reflects a deeper structural issue: a growing misalignment between data, method, and inference. When these three levels cease to be coherently articulated, replicability is no longer a property that scientific knowledge can sustain.

From this perspective, replicability is not an external criterion to be verified *post-hoc*, but an internal property of the research process itself. It is not something that is checked at the end; it is something that is built—or undermined—throughout each methodological decision. This Research Topic is positioned precisely at that level. It does not aim to provide isolated technical fixes, but to intervene at key points where contemporary research practices systematically produce results that may be formally correct, yet epistemologically fragile (Boulesteix et al., 2020; Open Science Collaboration, 2015).

The three premises underlying this Topic describe different facets of the same phenomenon. First, the increasing accessibility of advanced analytical methods has fostered an illusion of control, where methodological sophistication is implicitly assumed to compensate for weaknesses in design or data quality. Second, the expansion of research in digital environments has shifted control from design to analysis, often without sufficient reflection on the implications of this shift. Third, methodological knowledge—particularly that derived from Monte Carlo simulation studies—remains insufficiently translated into actionable decisions for applied researchers. Together, these dynamics create a scenario in which methodological complexity increases while inferential validity becomes progressively more uncertain.

The contributions included in this Research Topic allow us to delineate this problem with precision, but more importantly, they reveal that what is at stake is not a set of isolated errors, but recurrent patterns in the production of scientific knowledge.

Sorjonen and Melin provide a critique that should be considered foundational: the conflation of statistical modeling with explanation. Through a simulated reanalysis of longitudinal data, they demonstrate that prospective effects derived from cross-lagged models can reverse depending on model specification. This result underscores that statistical models do not uncover causal relationships; they instantiate them under a set of assumptions that are rarely subjected to sufficient scrutiny. The widespread practice of interpreting estimated parameters as if they were inherent properties of the phenomenon reflects a reification of the model that weakens inference. In this context, triangulation is not an optional refinement but a minimal requirement to ensure that conclusions do not depend exclusively on unexamined analytical choices.

Seaborn and Nakamura shift the focus to a prior, yet equally critical level: the production of data in digital environments. Their large-scale evaluation of online sampling shows that data quality cannot be taken for granted, even within widely used platforms. More importantly, it demonstrates that data are not neutral entities, but the outcome of participation dynamics, recruitment mechanisms, and design decisions that introduce specific structures of bias. The implication is direct: if the data-generating processes are not controlled or understood, the replication of analyses does not guarantee the replication of the phenomenon. Replicability therefore becomes a matter of controlling the conditions under which results are produced (Bentley et al., 2020; Peer et al., 2022).

In a complementary line, Mi et al. address one of the most demanding—and most frequently overlooked—conditions for valid inference: measurement equivalence. Their findings of partial invariance in stigma scales highlight that instruments are not transparent to the constructs they intend to measure. Assuming otherwise introduces an additional layer of uncontrolled error. Replicability cannot be reduced to the consistency of results; it requires ensuring that what is measured has the same meaning across contexts and groups (Vandenberg and Lance, 2000).

Fernández-García et al. locate the problem in a different, yet decisive domain: the translation of methodological knowledge into analytical decisions. Monte Carlo simulation studies have generated extensive evidence about the performance of statistical methods, yet their impact has been limited by how results are communicated. Summarizing outcomes through averages obscures condition-specific behavior and prevents applied researchers from identifying appropriate methods for their context. The introduction of Traceability Tables and the Variability Set shifts the focus from average performance to conditional behavior. This change makes methodological evidence directly usable (Morris et al., 2019).

Taken together, these contributions support a clear thesis: the replicability crisis is not the result of isolated failures, but of a systematic accumulation of unexamined decisions. Increasing analytical sophistication has not been matched by equivalent reflection on conditions of validity. As a result, behavioral science often produces findings that are formally correct but inferentially weak.

In response, this Research Topic advocates a conceptual reorientation: to understand replicability as a problem of distributed methodological responsibility. Method researchers must explicitly define the conditions under which their tools are valid. Applied researchers must critically evaluate their analytical decisions rather than relying on methodological complexity as a guarantee of validity.

Ultimately, replicability cannot be secured through checklists or increasingly complex techniques. It emerges from a scientific practice that maintains an explicit alignment between data, method, and inference. When this alignment is lost, replicability becomes an inevitable casualty.

This Research Topic contributes to restoring that alignment by identifying where failures occur and what changes are required. In a context where technical sophistication advances faster than methodological reflection, this effort is essential for sustaining a behavioral science that is cumulative, critical, and reliable.
